# “I’m not alone, my story matters”: Incarcerated women’s perspectives on the impact and acceptability of group psychotherapy involving imaginal exposure to sexual assault memories

**DOI:** 10.1186/s40352-021-00148-4

**Published:** 2021-09-30

**Authors:** Melissa J. Zielinski, Marie E. Karlsson, Ana J. Bridges

**Affiliations:** 1 Psychiatric Research Institute, University of Arkansas for Medical Sciences, 4301 W. Markham Street Little Rock, Little Rock, AR 72205 USA; 2 Department of Psychological Sciences, University of Arkansas, AR Fayetteville, USA; 3 Centre for Sexology and Sexuality Studies, Malmö University, Malmö, Sweden

**Keywords:** Incarcerated women, Exposure therapy, Group therapy, Trauma, Treatment acceptability, Prison, Imaginal exposure, Narrative exposure

## Abstract

**Background:**

Although it is clear that incarcerated women need access to effective therapies for trauma sequelae, some have argued that one of the most effective treatments – exposure therapy – should not be provided in carceral settings due to the presumed lack of safety and stability making such an intervention inappropriate. Group therapy, the typical mode of intervention in prisons, has also been presumed to be unacceptable for exposure-based processing due to assumptions that hearing others’ trauma narratives would be traumatizing and unhelpful to listeners. However, there is a lack of data to support either of the aforementioned assumptions. This study examined the acceptability of an exposure-based group therapy for women survivors of sexual violence who were currently incarcerated (*N* = 61) by asking women themselves about their experiences completing an exposure-based group therapy protocol (SHARE; *Survivors* *Healing from* *Abuse:* *Recovery through* *Exposure*) while incarcerated. We assessed women’s reasons for enrolling in the group, satisfaction with various therapy components (e.g., exposure, skill-building) and the treatment overall, and experiences of both sharing and listening to trauma narratives using a feedback survey that included a mix of multiple-choice and open-ended questions. Treatment dropout was examined as an additional index of acceptability.

**Results:**

Treatment completion was very high (88.8%). Nearly all women who completed the group reported that they would recommend it to other incarcerated women (96.7%, with the remaining 3.3% reporting “it depends”). Qualitative results revealed overwhelmingly positive feedback about the effect of the group and indicated that sharing and listening to trauma narratives in a group setting serve discrete but dually important functions. Specifically, women almost universally experienced listening to others’ trauma narratives (i.e., exposures) in the SHARE group context as helpful—making them feel less alone and normalizing their experiences. Sharing one’s own story primarily provided an emotional release and/or transformation (i.e., an intrapersonal rather than interpersonal function).

**Conclusions:**

Our findings challenge common concerns about the appropriateness of 1) prison as a context for trauma-focused treatments, including exposure and 2) sharing trauma narratives in a group setting. Unless empirical evidence demonstrating harm is uncovered, best practices for PTSD and other trauma-related sequelae—those recommended in reputable treatment guidelines and interventions like SHARE that incorporate components shown to be effective (e.g., cognitive challenging, exposure)—should be offered to incarcerated women as part of standard of care.

**Supplementary Information:**

The online version contains supplementary material available at 10.1186/s40352-021-00148-4.

## Introduction

Most women who are incarcerated—56-82% according to best estimates—have experienced sexual violence (Karlsson & Zielinski, 2020). Sexual violence is well known to increase risk for psychiatric disorders (Dworkin, 2018). Posttraumatic stress disorder (PTSD), major depressive disorder, and drug and alcohol use disorders are particularly common (Chen et al., 2010; Dworkin, 2018; Dworkin et al., 2017). Early exposure to sexual violence has even been hypothesized to be a pathway to prison for women due to its cyclical relations with substance use and mental illness, both of which place women at risk for arrest and incarceration (Lynch et al., 2017; Salisbury & Van Voorhis, 2009) and are linked to recidivism (Huebner et al., 2010; Sadeh & McNiel, 2015). Mental illnesses associated with trauma exposure are prevalent among incarcerated women: 15–29% meet criteria for PTSD, 10–25% meet criteria for major depressive disorder, 10–40% meet criteria for alcohol use disorder, and 53–78% meet criteria for drug use disorder (Karlsson & Zielinski, 2020). These prevalence estimates greatly exceed those found among women in the community, especially women without a history of rape (Pietrzak et al., 2011; Zinzow et al., 2012). For example, the aforementioned prevalence of PTSD among incarcerated women is 2–3 times the prevalence of PTSD found among community samples (Karlsson & Zielinski, 2020). Studies have also found that incarcerated women who have been sexually abused have worse physical and mental health compared to incarcerated women who have not been sexually abused (Aday et al., 2014) and that sexual abuse is linked to mental illness in this population (Lynch et al., 2017).

Yet, some scholars have argued that some of the most effective treatments for PTSD and related comorbidities—exposure-based therapies—are not safe to provide in carceral settings such as jails and prisons (Miller & Najavits, 2012; Wolff et al., 2009). For example, Wolff et al. (2015, p. 67) wrote that “the most robust evidence-base supports exposure therapy…this intervention is categorized as a second stage intervention, which must be delivered in safe and supportive environments…authoritative and punitive settings, like prisons, do not meet standards for exposure processing.” While these concerns appear face-valid given that victimization such as physical and sexual assaults certainly occurs in carceral settings (Wolff et al., 2009), there is a notable lack of empirical evidence to support the claim that exposure-based interventions cannot be safely provided to people who are currently incarcerated. Conversely, therapies that include exposure as a treatment component have proven effective in settings characterized by ongoing violence, limited privacy, and housing instability (Bass et al., 2013; Bolton et al., 2014; Grech & Grech, 2020; Mørkved et al., 2014; Thompson et al., 2018). Moreover, the implicit assumption that carceral settings are experienced as unsafe and unstable to *all* people that reside there is problematic. Numerous studies of incarcerated women have found that some women describe incarceration as a time of safety or respite compared to their lives in the community, and even describe health improvements (Alves et al., 2016; Bradley & Follingstad, 2001; Douglas et al., 2009; Goomany & Dickinson, 2015; Harner & Riley, 2013). Existing qualitative studies also provide proof that incarcerated women themselves find prison-based interventions for trauma sequelae acceptable (Abad et al., 2013; McCauley et al., 2020; Zielinski et al., 2020).

Clinician attitudes toward conducting imaginal exposure to trauma narratives (Feeny et al., 2003; Zoellner et al., 2011), including in therapy groups, are a further barrier to implementation of effective trauma-focused therapies in carceral settings. Arguments against conducting imaginal exposure in a group setting include that hearing others’ trauma narratives will be “re-traumatizing” (i.e., trigger distress and re-experiencing symptoms) for others in the group or may cause secondary traumatization (see Barreraet al., 2013 for a detailed review of concerns). Indeed, trauma therapies that were either intended for or can be delivered in group settings, like Seeking Safety (Najavits, 2002) and Cognitive Processing Therapy (Resick et al., 2017; Resick & Schnicke, 1992), have prohibited group members from sharing full trauma narratives in group even if sharing does occur in the context of other exercises (Beck & Coffey, 2005). Again, while these concerns appear face-valid given that acute distress is often experienced while revisiting memories of trauma in the context of exposure (Foa & Kozak, 1986), we are unaware of any evidence that doing imaginal exposure in the context of a therapeutic group has been associated with negative outcomes. Conversely, a meta-analytic review by Barrera, Mott, Hofstein, & Teng (2013) found that cognitive behavioral group therapies for PTSD, including those that contain in-session imaginal exposure, lead to large reductions in PTSD symptoms. Participants in groups that used exposure did not experience worsened outcomes compared to groups that did not use exposure; rather, the authors noted that “although comparison of effect sizes between treatments including any form of exposure…to those not including any form of exposure were not statistically significant (*p* = .166), treatments utilizing exposure yielded large effect sizes (ES = 1.32; 95% CI: 0.89 to 1.75), while the effect size estimates for treatments not including exposure were small to medium (ES = .49; 95% CI: -0.19-1.18)” (Barrera et al., p. 28). Moreover, the only study that had an explicit focus on asking exposure-based PTSD group members about their perspectives on the effectiveness and tolerability of such treatment found that group members experienced reliable reductions in PTSD symptoms, were highly satisfied with the treatment, found it both helpful and acceptable, and had very low dropout (5%) (Mott et al., 2013). Notably, group members also reported that their commitment to the group was a primary reason for remaining in treatment—suggesting that completing exposure therapy in a group may in fact be helpful for retention.

In summary, although some have expressed concerns regarding provision of exposure-based therapy to people who are incarcerated and in group settings (Barrera et al., 2013; Miller & Najavits, 2012; Wolff et al., 2009), there is a notable lack of data that empirically validates these concerns. To the contrary—the data that has been published suggests that people do benefit from PTSD treatment, even in settings characterized by conflict and instability, and that imaginal exposure can be delivered safely in groups (Barrera et al., 2013;Bass et al., 2013; Bolton et al., 2014; Grech & Grech, 2020; Mørkved et al., 2014; Thompson et al., 2018). Moreover, groups are feasible and familiar treatment modalities for under-resourced settings like jails and prisons, and interventions that cannot be delivered in a group format are unlikely to become widely available or adopted (Morgan & Flora, 2002). To our knowledge, no studies have surveyed incarcerated women themselves about their perspectives on the experience of receiving exposure therapy while incarcerated.

### The current study

The purpose of this study was to examine the acceptability of an exposure-based psychotherapy group for women survivors of sexual violence by asking incarcerated women themselves about the acceptability of offering exposure-based interventions (1) in prisons and (2) in the group setting. Acceptability refers to appraisals regarding the palatability of an intervention and is one of several key outcomes of implementation articulated in the implementation science literature (Proctor et al., 2011). Intervention acceptability is theorized to be critical to intervention adoption by providers (Weiner et al., 2017), but is also important at the patient level due to its relation to intervention engagement. For example, the Theory of Planned Behavior highlights that individuals’ attitudes about engaging in a behavior (such as attending a specific treatment) is one of the determining factors for whether they ultimately engage (Ajzen, 1991; Conner & Armitage, 1998). Studies of acceptability have commonly incorporated both self-report and behavioral measures and/or examined acceptability qualitatively (c.f. Akiyama et al., 2020; Johnson et al., 2019; Perry et al., 2019). Here, we sought to understand women’s perspectives given the importance of these constructs in such behavioral and implementation science theories and frameworks.

The evaluation used a feedback form that included a mix of multiple-choice and open-ended questions. Items focused on participants’ reasons for enrolling in the group, satisfaction with various therapy components (e.g., exposure, skill-building) and the treatment overall, and experiences of both sharing and listening to trauma narratives. Treatment dropout was examined as a behavioral index of acceptability. Consistent with our experiences leading and/or supervising SHARE, we anticipated that women would report having a positive experience and recommend it to other women. All other research questions were exploratory and/or descriptive given our goal of investigating women’s perspectives.

## Method

### SHARE intervention

SHARE is an 8-session group therapy for incarcerated women who are survivors of sexual violence (Karlsson et al., 2014, 2015, 2020; Zielinski et al., 2016, 2021). Each session is 1.5 h, summing to 12 h of therapy for each group. SHARE is facilitated by external volunteers who are licensed mental health professionals or clinical psychology doctoral students under the supervision of a licensed clinical psychologist. Partaking in SHARE is completely voluntary and participants are able to discontinue at any time. Group sizes are kept small (no more than 10 women) and confidentiality is heavily emphasized from the start of the group. The therapy consists primarily of psychoeducation, imaginal exposure, and group feedback; material on themes common to trauma (e.g., problems with safety and/or trust) are integrated when time allows. SHARE’s core component is imaginal exposure, a technique that is central to several evidence-based trauma-focused therapies (Foa et al., 2015) and is based on Emotional Processing Theory (Foa & Kozak, 1986). According to Emotional Processing Theory, in order to alter and overcome the fear networks associated with many trauma memories, individuals must emotionally activate and approach the memories. This is accomplished via imaginal exposure to the memory, which allows individuals to habituate to the memory and distinguish between the danger that was present at the time of the trauma and distress that may occur when a memory of that danger arises.

In SHARE, imaginal exposure to sexual assault memories occurs during 5 of the 8 treatment sessions (see Table 1). Each SHARE participant completes one imaginal exposure to a memory of sexual assault. Consistent with procedures for imaginal exposure in other protocols (e.g., Prolonged Exposure; Foa et al., 2015) women are asked to share their memory in narrative form, using as much detail as possible. We instruct participants to include details from their five senses, as well as what they can remember thinking and feeling at the time. They are encouraged to use behaviorally-specific language and to speak in present tense when describing the assaultive acts they experienced. They are also instructed to close their eyes if they are comfortable doing so to increase the emotional salience of the exposure. Group leaders help participants choose which memory to share about, if needed. Group leaders are the only ones who may “jump in” during the exposure. Typical reasons for doing so are to ask the sharer to add specific details, to go over part of a memory more slowly, or to ask questions intended to help the sharer challenge unhelpful thoughts about the assault. Consistent with Zoellner et al. (2011), our experience is that under-arousal during sharing is much more common than over-arousal; group leaders commonly work to help participants engage emotionally. While one participant is sharing, the rest of the group members are expected to listen without interrupting and to focus on being present with the sharer. After each exposure, if the sharer gives permission, group members are invited to give supportive feedback to the sharer. Instructions for providing support are discussed with the group prior to feedback. The group also processes the impact of the story on them and are assisted in selection of coping strategies as needed. A maximum of two participants share during each group session, as exposures typically take 20–40 min. See Table 1 for an overview of the focus of each SHARE group therapy session. Studies regarding the effectiveness of the SHARE intervention (Karlsson et al., 2014, 2015, 2020) and factors influencing SHARE’s implementation and sustainment (Zielinski et al., 2021) are available elsewhere. In short, SHARE has been shown to clinically and statistically improve symptoms of PTSD, depression, and anxiety and to meet a need for sexual violence intervention in women’s prisons.

**Table 1:** SHARE Group Outline

Session	Components	Therapeutic Tasks
1–2	• Establish group norms • Provide psychoeducation about trauma, trauma sequelae, and the rationale for exposure treatment • Teach coping techniques (e.g., grounding, deep breathing) for use during and outside of session	• Build rapport, trust, and safety • Enhance motivation • Build self-efficacy
3–7	• Conduct imaginal exposure • Give and receive supportive feedback following exposures • Identify and challenge common cognitive themes that emerge in trauma narratives.^a^ • Provide education on special topics related to sexual violence sequelae (e.g., healthy/unhealthy relationships)	• Approach rather than avoid distressing memories of sexual violence victimization • Facilitate emotional processing • Normalize experiences • Develop more balanced views of the traumatic event(s) and one’s role in it • Facilitate peer support
8	• Consolidate gains • Connect to local resources • Discuss relapse prevention	• Anticipate lapses • Provide hope for continued healing

^a^Occurs in Sessions 5–7 if time allows

### Setting

We conducted SHARE groups and this associated study at a minimum-security women’s community corrections center in the southern United States. The center has a maximum capacity of 120 residents, most of whom are non-Latina Whites who are incarcerated for drug-related crimes (i.e., possession, paraphernalia, distribution; ~ 70%). Failure to appear (~ 12%), financial fraud (~ 7%), and burglary or theft (~ 10%) are other common reasons for incarceration in the center. Sentences are typically either less than 1 year (33%) or 1–3 years (33%), although women are typically eligible for parole when they have served one-third of their sentence. SHARE has been offered as an optional program in the center continuously since 2012.

### Participants

Study participants were 80 incarcerated women who enrolled in 15 separate SHARE groups between March 2016 and May 2019. Each group had 4–8 participants and met for eight 1.5-h sessions, as is standard in SHARE. Participants were mostly White (90.2%, with 4.9% Black, and 4.9% Native American) and not currently married (73.8%), with an average age of 32.95 years (*SD* = 8.88, range = 19–57). These characteristics are consistent with the broader population at the facility where participants were incarcerated (described above). Most participants were also mothers of at least one child (90.2%; *M*_children_ = 2.49, range = 0–7). All women self-identified as having experienced sexual violence (as was required to participate in SHARE). The majority of participants screened positive for posttraumatic stress disorder (76%) and depression (67%) when completing assessment measures at pre-treatment.

Our sample size for most variables was 61 women who provided responses to a feedback form immediately after the last group therapy session (i.e., at the end of Session 8). See footnote 1 for more information about reasons for non-completion of the feedback form by 19 women due to a mix of group non-completion, researcher error, and non-availability or non-completion of research follow-up.^1^

### Procedure

All procedures were approved by the institutional review board at the University of Arkansas and by Arkansas Community Corrections. SHARE group participants were recruited via oral announcements during daily all-facility meetings at the corrections center. The announcement included a description of the structure and purpose of SHARE, behaviorally-specific examples of sexual violence, and examples of difficulties that are commonly experienced following sexual violence. Women were advised to submit a request to participate in the group to the center’s treatment coordinator if they were interested in joining an upcoming SHARE group due to self-identifying as having ongoing difficulties as the result of sexual violence they had experienced. There were no formal exclusion criteria (i.e., women were not screened by the group leaders or research team prior to participation).

All women who enrolled in SHARE were subsequently invited to participate in a study of the treatment’s acceptability and effectiveness. Study participation involved completing measures on paper on up to three occasions: pre-treatment (i.e., shortly before or during the first SHARE session), post-treatment (i.e., at the end of the last SHARE session), and follow-up (i.e., several months after the end of SHARE). During the informed consent process, which was conducted once per group either shortly before or during the first SHARE session, trained research assistants explained the study’s purpose and were available to answer any questions potential participants had. Participants were informed that they were free to skip over any items that they did not wish to complete and/or decide that they did not want to participate in the research study at any time. To maximize informed consent, we also used a double-consent procedure wherein participants had an opportunity to decline for their information to be used after completing all of the measures (i.e., they could change their mind and indicate that the researchers should not use their information on the last item in each assessment packet). All measures were taken out of the facility by the research team following completion to ensure participant privacy. In this study, we focus on presenting the results of a feedback survey (described below) that was administered during the post-treatment assessment.

### Measures

SHARE participants’ experience of and willingness to participant in exposure-based group therapy targeting sexual violence recovery was measured in two ways. First, we examined the percentage of treatment completion as a behavioral index of incarcerated women’s receptivity to SHARE treatment. Second, we assessed self-reported experiences of exposure-based treatment of incarcerated women who completed SHARE group via a group feedback form that was administered at the end of the treatment. The feedback form consisted of mutually exhaustive multiple-choice questions (e.g., yes/no) with open-ended follow-up questions and fully open-ended questions. Together, these indices comprised our approach to examining SHARE’s acceptability. Each section of the group feedback form is described in more detail below.

#### SHARE completion/dropout and attendance rates

Completion and dropout rates for SHARE group therapy were assessed by calculating the percentage of women in each category from the total number of participants who enrolled in SHARE during the study period. Criteria to be considered a completer included having attended a minimum of five treatment sessions and having shared one’s own trauma narrative in the group (i.e., completed the individual imaginal exposure). Attendance rates were calculated based on group leaders’ records of participant attendance (i.e., number of sessions attended out of 8 total possible).

#### SHARE feedback form

The SHARE feedback form was created for this study. Full text of all items is available in Additional file 1. The form inquired about the major areas described below.

##### Reasons for enrolling in group

Participants were asked to rate the influence of five potential reasons for enrolling in SHARE on a scale from 1 (*Not at all*) to 5 (*Extremely*). Items assessed potential internal motivators (“I wanted to talk to someone about the sexual violence I have experienced” and “I wanted/needed help with some of the outcomes related to the sexual violence I have experienced (e.g., anxiety, anger, distrust)”) and external motivators (being encouraged to sign up by each of the following: previous group participants, mental health providers at the facility, and staff members at the facility). Participants were also given the option to qualitatively describe and rate any motivating reasons not listed.

##### Dropout considerations

Participants were asked if they had considered dropping out (yes/no). If the participant had considered dropping out, she completed three follow-up questions: (1) “When did you consider dropping out?”, (2) Why did you consider dropping out?”, and (3) “What made you stay in this group?”. All participants were also asked, “If some of your group members did not complete the group, why do you think they dropped out?”

##### Helpfulness of treatment elements

Participants were asked to rate the helpfulness of 11 treatment elements included in SHARE group therapy on a scale from 1 (*Not at all*) to 5 (*Extremely*). Full text of all items is included in Table 3 of the *Results* section. Women were also asked, “Did any part of the program have a negative impact on you?” (yes/no). If the participant indicated that the program had had any negative impact, she was asked to provide details.

##### Experiences sharing and listening to trauma narratives

Participants were asked three questions related to their experiences sharing and listening to trauma narratives. First, they were asked the open-ended items “What impact did sharing your story in the group have on you?” and “What impact did hearing the other group participants’ stories have on you?” Participants were also asked about the degree of similarity between their own trauma narrative and that of other members of their group when considering details such as age of assault, perpetrator characteristics, and duration of trauma. This item was included so that we could consider whether perceived degree of similarity may be related to other responses.

##### Overall treatment acceptability

Participants responded to two final questions related to their overall perceptions of the group. First, they were asked “Would you recommend this treatment to other women who have experienced sexual assault, violence, or abuse?” Response options were “No,” “Yes,” and “Depends,” with a subsequent request for a narrative description of the reason for selecting that answer. Second, they were asked whether they thought that doing the treatment individually, instead of in a group, would be more helpful, less helpful, or “it depends.” This was meant to help us assess preferences for an alternate modality. As before, participants were asked to provide a narrative description of the response option they selected.

### Analytic approach

Two members of the research team (the first and second authors; MZ and MK) performed thematic analysis of all qualitative data that was generated in response to the feedback form. The data was entered in to a database separately by feedback item. The first author (MZ) developed the preliminary descriptive themes for each item after reviewing all of the data for that item. This process was inductive and iterative, and was undertaken with a realist lens. After the preliminary codebook was developed, MZ and MK independently coded all responses and met to discuss and resolve discrepancies. Discrepancies were solved through discussion and consensus building, and minor revisions to the wording of several themes were made collaboratively during discussion. This process aligns with the steps of thematic analysis described by Braun and Clarke (2006).

## Results

### Completion/attendance rates

Of the 80 women who enrolled in SHARE, 71 completed the 8-session group – a 88.8% completion rate.^2^ Most treatment completers attended all 8 sessions (71.8%, or *n* = 51) or missed only one session (21.1% or *n* = 15). Only five participants attended fewer than 7 of the 8 sessions.

### Reasons for enrolling in SHARE

Of the five researcher-provided reasons for enrolling in SHARE, wanting/needing help with outcomes of sexual violence (e.g., anxiety, anger, distrust) was rated most highly (*M* = 4.05, *SD* = 1.08). Being encouraged to take the group by a facility counselor (*M* = 3.39, *SD* = 1.64) and wanting to talk about the sexual violence experienced (*M* = 3.27, *SD* = 1.23) were the next most highly rated reasons. Twelve women wrote in “other” responses which centered around themes of a desire for change (*n* = 8; e.g., “To get out of the cycle I was in” and “I wanted to prevail”), a general positive perception of group (*n* = 3; “I thought group therapy would help,” and “I knew in my heart when I first hear[d] of it that I needed to take it”), and inspiration from others (e.g., *n* = 2; “My mother took this group when she was in here and it helped her a lot”).

### Dropout considerations

Many participants reported that they had considered dropping out of the group (39.3%; *n* = 24). Themes that emerged from qualitative responses to the question, “Why did you consider dropping out?” and “What made you stay?” and corresponding illustrative quotes are summarized in Table 2. All but one participant answered both items (this participant answered neither open text item). Most participants gave responses that fit in to one theme; however, some gave a response that fit in to more than one theme (*n* = 8 for drop out reasons and *n* = 1 for reasons for staying). 

The most common reason women provided for considering dropping out of SHARE treatment was fear (*n* = 12). The focus of the fear varied between respondents, but included things like fear of their confidentiality being broken, fear of sharing their story with others, and fear of the discomfort they anticipated would come with sharing. Only one participant reported not wanting to share as a factor in considering dropout in a way that did not specifically reference fear. Interpersonal concerns (*n* = 9) and emotions other than fear (*n* = 6) were also common amongst those who considered dropping out. 

Women’s reasons for staying in the group despite considering dropping out were more variable and fit into four broader themes: 1) positive anticipatory emotion, 2) the group environment, 3) personal empowerment or determination, and 4) desire for change. One participant also pointed to the role of her facility counselor in encouraging her to trust the group leaders, something she was scared to do.

**Table 2:** Themes and Illustrative Quotes for Dropout Items

Reasons for Considering Dropping Out		Reasons for Deciding to Stay	
Theme	Illustrative Quotes	Theme	Illustrative Quotes
1. Fear (*n* = 12)	• “Scared to tell others my story.” • “I was afraid I was going to stay angry and relapse”^a^ • “I was overwhelmed with knowing that I would soon have to tell my story. The fear of exposure was almost too much.”	1. Positive anticipation (*n* = 6)	• “Because I knew it would help me.” • “Maybe it will help and it did a lot.”
2. Interpersonal concerns (*n* = 9)	• “I was scared that the group would not keep my story to the group.”^a^ • “Because I had trust issues.” • “I didn’t want to be judged by others.”	2. Group environment (*n* = 5)	• “The support and encouragement of the group.” • “I just had a trusting easy feeling after I talked to [the group leaders].”
3. Emotions besides fear (*n* = 6)	• “I knew it was going to hurt” • “Embarrassment, shame” • “Because I was feeling feelings I haven’t felt in a long time”	3. Personal empowerment/ determination (*n* = 5)	• “I am strong and a survivor.” • “Dedication to myself and my recovery and victory over my experiences.”
4. Miscellaneous responses (*n* = 4)	• “Thought that my story wasn’t important.” • “Too much was going on in my life at the moment.”	4. Desire for change (*n* = 5)	• “I needed to get it off my shoulders.” • “Fear of not changing, fear of myself.”
		5. Miscellaneous responses (*n* = 3)	• “I made a commitment plus I think it helped me.”

Note: ^a^ = multiple themes were judged as applicable to this participant’s response and it was coded in multiple categories

All participants, regardless of whether they considered dropping out, were asked for their thoughts on why group members who dropped out did so (if applicable, since some groups did not experience any drop out). Participants (*n* = 31) perceived that people who dropped out did so either due to interpersonal factors (*n* = 9; e.g., “They were not respectful of other people,” “Because they felt uncomfortable in our group”) or because they were not ready (*n* = 8; e.g., “They weren’t ready” and “Because they were scared and not ready for it maybe”). Fear (*n* = 8) and emotions besides fear (*n* = 5) such as shame and being overwhelmed were mentioned as possible reasons; however, women also reported that non-attendance due to personal choice as well as structural barriers to attendance led to dropout (*n* = 6; “Missed too many groups,” “Because they left and went home,” and “Because they didn’t want to be in the group during their down time”).

### Helpfulness of treatment elements

All of the 11 researcher-provided treatment elements that women rated had a mean helpfulness score above “4” (“Quite a bit” helpful), suggesting that participants found each of the group elements to be therapeutic. “Being in a group of women who had all also experienced sexual assault or abuse” was rated as most helpful, and likely served a normalization function given the occurrence of exposure within the group (see next section). Provision of coping skills had the lowest mean score. Mean helpfulness scores for sharing and listening to others’ stories were identical, though the qualitative responses presented in the next section suggest that these treatment elements serve discrete functions. See Table 3 for ratings for all treatment elements.

**Table 3:** Helpfulness Ratings of SHARE Treatment Elements

Rank	Treatment Element	Category	M*	*SD*
1	Being in a group of women who had also all experienced sexual assault or abuse	Normalization facilitated by exposure	4.71	0.53
2	Feedback/Support from the group facilitators after you shared your story	Exposure	4.68	0.71
3	Receiving information about the importance of sharing our stories about sexual assault (e.g., why it can be helpful to talk about your memories)	Exposure	4.61	0.67
4	Hearing other people’s stories about their sexual assault experience(s)	Exposure	4.59	0.65
4	Sharing your story about your sexual assault experience(s)	Exposure	4.59	0.65
6	Feedback/Support from the group members after you shared your story	Exposure	4.53	0.80
7	Receiving information about sexual violence, assault, and abuse.	Psychoeducation	4.51	0.80
8	Discussing different topics related to sexual violence (e.g., trust, intimacy, how to talk to your children about sexual abuse, healthy/unhealthy relationship signs)	Psychoeducation	4.49	0.77
9	Providing feedback/support to other group members after they shared their stories	Exposure	4.48	0.82
10	Receiving information about PTSD, depression, anxiety, and other common mental health problems following sexual assault	Psychoeducation	4.44	0.86
11	Coping techniques (e.g., grounding, breathing)	Coping	4.41	0.83

Note: Helpfulness ratings range from 1 (*Not at all helpful*) to 5 (*Extremely helpful*)

### Experiences sharing and listening to trauma narratives

#### Impact of sharing

Participants’ responses to the question “What impact did sharing your story in the group have on you?” were varied but almost universally positive (see Table 4). The dominant theme within the responses was that sharing one’s experience of sexual violence victimization via imaginal exposure in group lead to emotional release and/or transformation. There were several more infrequent themes, including: 2) changed view of other people and/or increased connection with others, 3) transformed self-view, 4) challenged self-blame, 5) general positive impact, and 6) was hard. Most participants gave responses that fit in to one theme; however, some gave a response that fit in to more than one theme (*n* = 8). One person gave a response that was too incomplete to be categorized.

**Table 4:** Impact of Narrating Memories of Sexual Assault/Abuse (i.e., Completing Imaginal Exposures) in Group

Theme	Illustrative Quotes
1. Emotional release and/or transformation (*n* = 25)	• “It’s like a weight has been lifted off my shoulders” • “It made me feel free from the prison I was living inside my head” • “Sense of relief from holding it in for 17 years” • “Releasing all the pain, anger, and guilt has given me a sense of freedom I have never had. A great weight has been lifted from inside me. Thank you.” • “Released the shame holding me down.” • “I have slept better in the last month and a half that I have in twenty-five years and I have got a new peace about me and in my life.”
2. Changed view of people and/or increased connection with others (*n* = 9)	• “It helped me to realize that not everyone is the same. Loosen up and learn to trust.” • “It was amazing to know that people believed me and others have understood what I went through.” • “I believe it helped me in a way sharing alone never has.”
3. Transformed view of oneself (*n* = 7)	• “I had a major breakthrough. I peeled the mask off.” • “Tremendous – believe that it was my gateway to becoming a victor, instead of a victim over my abuse.” • “It made me…realize that I am a good and forgivable person….” • “An extremely positive one. I am beautiful and enough.” • “A different perspective of myself”
4. Challenged self-blame beliefs (*n* = 6)	• “…made me see it wasn’t my fault.” • “It made me accept the fact it wasn’t my fault…”
5. General positive impact (*n* = 6)	• “It helped me in many ways.” • “I see that the group helped me a lot.” • “A huge impact. I never thought I could look at my past the way I am able to do today.”
6. Was hard (*n* = 4)	• “It felt horrible at first. But now I understand that there were emotions I didn’t know I had inside that intertwine with problem areas of my life.” • “It was hard to actually open up and share how I felt about my abuse.” • “It was hard for me but it helped by hearing three other sisters share first. It made it a lot easier for me.”

#### Impact of listening

Participants’ responses to the question “What impact did hearing the other group participants’ stories have on you?” largely centered around one single theme—realizing that one was not alone (*n =* 41; see Table 5). In many cases, participants reported feeling a positive transformation in how they view their feelings, worth, or personhood as being linked with realizing that others had gone through the same experiences. A large portion of these responses (78%) included the words “not alone” directly. A second theme focused on other types of positive interpersonal impacts such as feeling more able to trust, feeling more connected to others, and that other people sharing their stories made one feel personally more able to share. Only one participant wrote that listening to others’ stories was distressing; she reported using the grounding skills that were taught prior to sharing during the group (see last quote in the Miscellaneous section of Table 5). Most participants gave responses that fit in to one theme; however, some gave a response that fit in to more than one theme (*n* = 5). There were 5 missing responses.

**Table 5:** Impact of Listening to Sexual Assault/Abuse Memories (i.e., Imaginal Exposures) in Group

Theme	Illustrative Quotes
1. Realized I’m not alone (*n* = 41)	• “That they hurt just like me. That I’m not alone.” • “Huge. Hearing other women feeling the same.” • “To know I’m not alone.” • “Realizing I’m not alone and that there are others out there who has been through similar traumatic events and that people are willing to help.” • “It helped me see that I’m not alone and I have a right to my feelings.” • “That it’s okay to feel the way I do. I’m not alone, my story matters.”
2. Other positive interpersonal impact (*n* = 12)	• “Intimacy of closeness with another” • “Taught me to trust” • “A sense of closeness, understanding.” • “It really impacted me all of the sisters stories. It made me feel comfortable in sharing and my trust has really grown.” • “Strong impact. Helped in making me more comfortable in sharing my story.” • “Empowering. Welcoming.”
3. Miscellaneous (*n* = 7)	• “It helped me identify some of the things I was blocking out from my own experiences.” • “It touched my heart deeply. I felt some empathy for them.” • “I was emotional, it was hard listening to their stories. I know I found myself counting or trying to focus on something when it became very overwhelming.”

### Overall treatment acceptability

Overall treatment acceptability was assessed with three questions: 1) “Would you recommend this treatment to other women who have experienced sexual assault/violence/abuse? Why or why not?”, 2) “Did any part of the program have a negative impact on you?”, and 3) “Do you think that doing this treatment individually, instead of in a group as we did, would be more helpful or less helpful? Please explain your answer.”

#### Assessing whether women would recommend SHARE to others

**Fig. 1:**
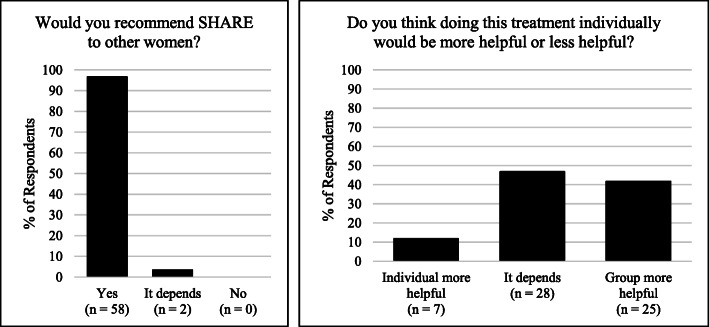
Participants’ Willingness to Recommend SHARE and Receptivity to Group Modality

When asked if they would recommend the treatment to other women, nearly all women indicated that they would (see Fig. 1). When asked the reason they would recommend SHARE, women largely cited the positive impact that the group had had on them and/or their feelings of connection with other people—aligning with a combination of the feedback that was given in response to the items that inquired about the impact of sharing and listening to exposures. Illustrative quotes included statements such as: “because this group is a healing group,” “it helps you learn to trust,” “it lightened my load and gave power back,” “because I don’t feel so alone, and being able to talk and deal with my feelings and feeling accepted by other women,” “it’s helped me sleep better,” “I wish I would have been able to do this when I was a kid. It would have saved me a lifetime of problems,” and “because it probably saved my life.” The two people who marked “it depends” in response to this item did not provide an open text response.

#### Alternative modality preference

When asked if they believed that individual therapy or group therapy would be more helpful, most participants indicated either a preference for group therapy or feeling ambivalent (see Fig. 1). When asked the reason for their selection of preferred therapy mode, people who chose group as preferable often described it being helpful to listen to other women’s experiences and feedback. Several described the group modality being helpful in building trust. People who chose “it depends” often described seeing value in both modalities as well as being open to doing a mix.

#### Assessing possible negative impact

Given the concerns that have been raised about providing trauma-focused interventions to people who are incarcerated, participants were asked “Did any part of the program have a negative impact on you?” Of the 58 women who provided responses to this item, 7 participants (11.5%) marked “yes.” However, qualitative responses revealed that only four participants (6.6%) reported symptom exacerbation (“brought out pent up anger,” “nightmares, but they are easing and confusing emotions are starting to make sense,” “kind of fueled my fire against [perpetrator],” and “I started to get rude to people again to push them away…I had to check myself”). Two participants (3.3% reported conflict with either a group member or group leader) and one participant simply stated “paperwork” (1.6%). However, nearly all of the participants who reported some negative impact (6 out of 7) also said that they would recommend SHARE to other women. The remaining participant marked “it depends.”

## Discussion

While there is a long and rich history of investigation into group therapy to assist with outcomes of trauma sequelae such as PTSD (Sloan & Beck, 2016), including therapies that integrate narrative exposure (see Barrera et al., 2013 and Sloan et al., 2013 for meta-analyses), extant research has been almost exclusively focused on assessing effectiveness by examining symptom changes, and not on group members’ experiences and perspectives completing these interventions (though see Mott et al., 2013 for an exception). This is true among the many studies that have examined approaches to trauma treatment for women who are currently incarcerated—most of which are coping-focused, supportive only, and/or primarily psychoeducational (see Emerson & Ramaswamy, 2015 and King, 2017 for recent reviews) and do not include treatment elements common to evidence-based therapies for trauma recovery, such as exposure.

Here, we examined incarcerated women’s experiences completing an exposure-based group therapy for sexual violence recovery. This study, to the authors’ knowledge, is the first to examine the acceptability of an exposure-based group therapy for women survivors of sexual violence who were currently incarcerated. Importantly, we placed women’s own experiences at the center of the investigation and we used quotes frequently throughout the manuscript to highlight their voices. Our results revealed that women signed up for SHARE—an exposure-based therapy—primarily because they wanted help with symptoms. Encouragement from others (e.g., facility counselors, staff peers) was also influential. Many women had considered dropping out at some point during the group, often due to emotions like fear and shame, though sometimes due to interpersonal concerns related to confidentiality, judgment, or trust. However, 88.8% of the women who originally enrolled completed SHARE. These results are consistent with Mott et al.’ (2013) study of Veterans’ perspectives on completing group-based exposure therapy which found that 78% of the sample had considered dropping out but 95% completed the intervention. Average dropout rates in community-based PTSD treatment are approximately 20% overall, and estimated to average 36% in trauma-focused interventions such as SHARE per meta-analysis (Imel et al., 2013). The lower dropout rate evidenced in SHARE may be a promising index of the acceptability and/or due to fewer barriers to attendance while incarcerated and/or participation in programming being rewarded. To the authors knowledge there has not been a review of dropout rates for in-prison programming that we can compare our results to; however, SHARE completion is higher than the median group completion rate found in the 14 unique studies of prison-based therapy groups for trauma survivors that were included in recent review papers on this topic (i.e., Emerson & Ramaswamy, 2015; King, 2017).^3^ Importantly, this finding is not due to coercion, as SHARE is a voluntary group. Moreover, even women who considered dropping out voiced strong reasons to stay including wanting help/knowing that they needed it and finding the group atmosphere healing. The latter finding was also consistent with Mott et al.’ (2013) study in which group participants indicated that social factors were important determinants of their decision to remain in the group.

Contrary to concerns that prisons do not meet standards for exposure processing (Wolff et al., 2015), nearly all women (96.7%) reported that they would recommend SHARE to other incarcerated women. The qualitative data made clear that women almost universally experienced listening to others’ trauma narratives (i.e., exposures) in the SHARE group context as helpful, making them feel less alone, more connected, and serving a normalization function. This finding is consistent with Yalom’s conceptualization of universality as a primary curative factor in group psychotherapy (Yalom & Leszcz, 2005). Sharing one’s own story seemed to have an equally important but discrete function as women highlighted this aspect of the group primarily provided an emotional release and/or transformation (i.e., an intrapersonal function). Importantly, few participants reported any source of negative impact from the group, with most being for reasons other than symptom exacerbation (though this was reported by a very small number of women).

We also considered that even if group was experienced positively, it was possible that women would prefer individual therapy over group. This was not the case; most women in our sample reported either preferring group or feeling equivocal (i.e., seeing some advantages to group as well as some advantages to individual interventions). This finding is important because many correctional facilities are characterized as high need but under-resourced settings where group treatment is likely to be the only feasible option for broad impact (Morgan & Flora, 2002). Knowing that group treatment is acceptable, and often even preferable, for women who have experienced sexual assault is encouraging. Since the majority of women who are incarcerated have been victims of sexual assault at some point in their lifetime (Karlsson & Zielinski, 2020), and given the relation between untreated trauma sequelae and likelihood of recidivism (Lynch et al., 2017), expanding access to evidence-based, first-choice treatments like exposure therapy is a priority.

### Strengths and limitations

The study’s strengths should be considered in light of its limitations. To our knowledge, this is the first qualitative study of the acceptability of an exposure-based group treatment for sexual assault victimization in incarcerated women. Given the often-cited concerns about the potential harms and pitfalls of offering such treatments in prisons (Miller & Najavits, 2012; Wolff et al., 2009), hearing from actual treatment participants is invaluable. On the other hand, the study included only the perspectives of women who had successfully completed SHARE. This study therefore cannot speak to the perspectives that women who never signed up for SHARE may have had about the intervention. Also, women who chose not to enroll after completing baseline measures or who dropped out of treatment did not provide input. While the majority of women did complete treatment, the nearly 12% who did not may have had notably different experiences with SHARE or have experienced different barriers to completion. Understanding their perspective, including whether there are particular variables that make someone a good candidate for group treatment, would be a helpful direction for future research. It may be that people who dropped out differed in important ways from people who remained in treatment; knowing this could help therapists screen potential group participants to increase the likelihood that SHARE or similar groups are appropriate and meet the need of each particular person at that particular time. Because data were only obtained from treatment completers, it is also possible they were experiencing effort justification, a cognitive strategy to reduce dissonance that involves increased valuing of an outcome where one had to work hard to obtain the outcome (Aronson & Mills, 1959). It may be that, having revealed a traumatic, possibly shame-ridden memory of assault or abuse in front of a group of peers, women emerged from the group valuing the group experience more than they would have if they had not had to work so hard or make themselves as vulnerable. On the other hand, exposure therapy is precisely about facing fears (Foa & Kozak, 1986) and group therapy by nature involves being vulnerable in front of others; both provide corrective emotional experiences (of overcoming fear; of being seen as acceptable and valued by others).

Participants in the study were from a single correctional facility in Arkansas; it is not clear if results would generalize to other settings. The facility houses women convicted of non-violent offenses and women are typically incarcerated for brief (< 2 year) periods of time. The facility also includes numerous therapeutic programs and focuses extensively on rehabilitation. Indeed, women typically arrive at group with a solid foundation in cognitive and behavioral coping strategies. A recent retrospective process that examined determinants of the implementation and sustainment of SHARE in this facility found that both the therapeutic nature of the facility and strong leadership support for the intervention positively influenced sustainability (Zielinski et al., 2021). Groups were also fully voluntary and group leaders were clinical psychology doctoral students who volunteered their time; therefore, none had dual relationships with the group participants (e.g., both therapist and guard). It is likely that these factors positively influenced the acceptability of SHARE, enhancing women’s willingness to disclose traumatic experiences without fear of future reprisal (at least from group leaders; the fear of ruptures in confidentiality remain present for group participants and is addressed directly in the first few sessions). A larger trial with a larger sample size and number of sites is warranted to examine generalizability given the relatively small sample in this study. More in-depth qualitative work would also be valuable as the data in this study were drawn from written accounts and thus we were unable to explore the themes would in greater depth or clarify meaning. Furthermore, the sample was heterogeneous with regard to sexual violence victimization experiences and current psychiatric symptoms. It is not clear if such heterogeneity helped increase the value of SHARE to participants or whether more homogenous groups would have resulted in even higher indices of acceptability. Future studies could explore the extent to which ratings of shared narratives’ similarities to one’s own relate to symptom changes across treatment.

## Conclusions

Together, the results of this study indicate that incarcerated women experience SHARE, an exposure-based group therapy for sexual violence recovery, as both acceptable and beneficial. This point is underscored by both women’s behavior (i.e., low dropout) and their written feedback. Our findings challenge common concerns about the (in)appropriateness of (1) prison as a context for trauma-focused treatments, including exposure, and (2) sharing trauma narratives in a group setting. Indeed, high completion rates in combination with high intervention acceptability may point to incarceration as an especially important time to at least offer women an opportunity to complete trauma-focused interventions. Future work should continue to explore the effectiveness and implementation of brief, exposure-based group treatments in prisons and other high need settings unless empirical evidence demonstrating harm is uncovered.

## Supplementary Information

**Additional file 1.**  SHARE Feedback Form.

## Data Availability

Not applicable.

## Footnotes

1 Of the 19 SHARE participants who did not complete the feedback form, less than half were SHARE non-completers (*n* = 8). Reasons for missing measures for the remaining 11 participants included that the feedback form was accidentally not included in post-assessment measure packets for one SHARE group (*n* = 5), that some SHARE completers had to miss Session 8 (i.e., when measures were administered; *n* = 4), and measure non-completion among SHARE completers who were present at Session 8 (*n* = 1 missed or chose not to complete the feedback form but completed other assessment measures; *n* = 1 was present at session 8 but needed to leave early or elected not to complete any study measures).

2 One participant who has been counted as a completer discontinued the first group she enrolled in, but enrolled again in the next group and completed. Calculating the completion rate at the group-level across the fifteen included groups (rather than at the individual level, as above) results in a 88.5% completion rate average per group.

3 These two review articles covered 14 unique studies of prison-based group interventions intended for trauma survivors. Of these 14 studies, 7 did not report information that would be needed to calculate dropout. Of the remaining 7 studies, treatment completion ranged from 54 to 96%, with a median of 83% completion.
