# Supplementary material for: “I’m not alone, my story matters”: Incarcerated women’s perspectives on the impact and acceptability of group psychotherapy involving imaginal exposure to sexual assault memories
Source: Health Justice. 2021 Sep 30;9:25. doi: 10.1186/s40352-021-00148-4 (PMC8482612; doi:10.1186/s40352-021-00148-4)
Supplement: Supplementary file 1 — Additional file 1. SHARE Feedback Form. [file 40352_2021_148_MOESM1_ESM.docx]

Feedback about the Sexual Violence Therapy Group

1. Please use the following scale to indicate how much each option affected your decision to participate in this group treatment:

**Not at all A little Moderately Quite a bit Extremely**

1. **2 3 4 5**

_____I wanted to talk to someone about the sexual violence I have experienced

_____I wanted/needed help with some of the outcomes related to the sexual violence I have experienced (e.g., anxiety, anger, distrust)

_____Previous group participant(s) encouraged me to sign up

_____A mental health provider at the facility encouraged me to sign up (e.g., counselor)

_____Staff member (e.g., not a mental health provider) at the facility encouraged me to sign up

_____Other reason: *Please specify*:____________________________________________

1. Did you consider dropping out of the group?

_____Yes

_____No

**If Yes:**

When did you consider dropping out (*Select all that apply*)?

_____Before the first session

_____After the first session

_____After the second session (before we started sharing our stories)

_____Right before it was my turn to tell my story

_____Right after I had shared my story with the group

_____Half way through (sessions 3-5)

_____Towards the end (sessions 6-8)

_____Other: *Please specify*:____________________________________________

Why did you consider dropping out?

What made you stay in this group?

1. If some of your group members did not complete the group, why do you think they dropped out?

1. How much did this group help you in the following areas (circle the number that best reflects the amount of change):

|  | **Much worse** |  |  | **No change** |  |  | **Much improved** |
| --- | --- | --- | --- | --- | --- | --- | --- |
|  |  |  |  |  |  |  |  |
| Anger | -3 | -2 | -1 | 0 | +1 | +2 | +3 |
|  |  |  |  |  |  |  |  |
| Trust | -3 | -2 | -1 | 0 | +1 | +2 | +3 |
|  |  |  |  |  |  |  |  |
| Guilt | -3 | -2 | -1 | 0 | +1 | +2 | +3 |
|  |  |  |  |  |  |  |  |
| Physical health | -3 | -2 | -1 | 0 | +1 | +2 | +3 |
|  |  |  |  |  |  |  |  |
| Mental Health | -3 | -2 | -1 | 0 | +1 | +2 | +3 |
|  |  |  |  |  |  |  |  |
| Spirituality | -3 | -2 | -1 | 0 | +1 | +2 | +3 |
|  |  |  |  |  |  |  |  |
| Social support from the group members outside of group sessions | -3 | -2 | -1 | 0 | +1 | +2 | +3 |
|  |  |  |  |  |  |  |  |
| Relationships with friends (excluding your relationships with the other group members) | -3 | -2 | -1 | 0 | +1 | +2 | +3 |
|  |  |  |  |  |  |  |  |
| Relationships with partners | -3 | -2 | -1 | 0 | +1 | +2 | +3 |
|  |  |  |  |  |  |  |  |
| Relationships with family members | -3 | -2 | -1 | 0 | +1 | +2 | +3 |

1. Please use the following scale to indicate how helpful each component of the group was for you:

**Not at all A little Moderately Quite a bit Extremely**

**1 2 3 4 5**

_____ Receiving information about sexual violence, assault, and abuse

_____ Receiving information about PTSD, depression, anxiety, and other common mental

health problems following sexual assault

_____ Receiving information about the importance of sharing our stories about sexual

assault (e.g., why it can be helpful to talk about your memories)

_____ Coping Techniques (e.g., grounding, breathing)

_____ Sharing your story about your sexual assault experience(s)

_____ Hearing other people’s stories about their sexual assault experience(s)

_____ Feedback/Support from the group members after you shared your story

_____ Feedback/Support from the group facilitators after you shared your story

_____ Providing feedback/support to other group members after they shared their stories

_____ Discussing different topics related to sexual violence (e.g., trust, intimacy, how to talk

to your children about sexual abuse, healthy/unhealthy relationship signs)

_____ Being in a group of women who had also all experienced sexual assault or abuse

1. Did any part of the program have a negative impact on you?

_____No

_____Yes

**If yes: Please explain:**

1. What impact did sharing your story in the group have on you?
2. What impact did hearing the other group participants’ stories have on you?
3. How similar were the stories you heard other women tell to your own (e.g., details such as your age when the sexual assault occurred, perpetrator’s characteristics, and duration of the trauma)?

_____All were very similar

_____Most were very similar

_____About half were similar to my own

_____Most were *not* similar to my own

_____None were like my own

1. Would you recommend this treatment to other women who have experienced sexual assault/violence/abuse? Why or Why not?

_____No

_____Yes

_____Depends

**Please explain your answer:**

1. Do you think that doing this treatment individually, instead of in a group as we did, would be more helpful or less helpful?

_____More helpful

_____Less helpful

_____Depends

**Please explain your answer:**
